# Correlation of Anti-*Salmonella* Antibodies Between Serum and Saliva Samples Collected From Finisher Pigs

**DOI:** 10.3389/fvets.2019.00489

**Published:** 2020-01-10

**Authors:** Alessia De Lucia, Shaun Cawthraw, Rob Davies, Richard P. Smith, Carlo Bianco, Fabio Ostanello, Francesca Martelli

**Affiliations:** ^1^Department of Veterinary Medical Sciences, School of Agriculture and Veterinary Medicine, Bologna, Italy; ^2^Animal and Plant Health Agency (APHA), Bacteriology Department, Addlestone, United Kingdom; ^3^Animal and Plant Health Agency (APHA), Epidemiology Department, Addlestone, United Kingdom; ^4^Animal and Plant Health Agency (APHA), Pathology Department, Addlestone, United Kingdom

**Keywords:** *Salmonella* antibody, saliva, oral fluid, serum, pigs, ELISA

## Abstract

Saliva samples obtained by using absorptive devices, can provide an alternative diagnostic matrix to serum for monitoring disease status in pigs. The aim of this study was to investigate the correlation of anti-*Salmonella* antibodies between serum and saliva samples collected from pigs. Twenty individual paired serum and saliva samples were collected from a single farm. Anti-*Salmonella* IgG was detected in individual serum samples using a commercial *Salmonella* ELISA kit, validated for sera. The same kit was used with a protocol modified by extending incubation time and increasing temperature to test individual saliva samples. Anti-*Salmonella* IgG antibodies in pig saliva were always detected at a lower level than in the matching serum samples. A correlation (*rho* = 0.66; *p* = 0.002) and a moderate agreement (*K* > 0.62 *p* = 0.003) was found between individual *Salmonella* IgG in serum and saliva samples. Both correlation and the agreement levels are moderate. The size of this investigation was small, and further studies are necessary to further confirm these findings. The results of this work provide some evidence that saliva samples have the potential to be used for the diagnosis of *Salmonella* infection in pig farms.

## Introduction

*Salmonella* is an important foodborne pathogen and the consumption of contaminated pork meat is one of the major sources of human outbreaks (1). In the latest Europe-wide survey, the prevalence of *Salmonella* in United Kingdom pigs was amongst the highest in Europe (2). Surveillance in pig herds is limited by the cost-effectiveness and efficiency of sampling methods (3). Disease monitoring often involves blood sampling for serological assessment, or environmental sampling (for example floor fecal swabs) for bacteriological culture, which are costly to the farmer due to veterinary fees (blood sampling) or require several days for a result (bacteriology) (3, 4). In the last decade, oral fluid (OF) diagnostic technology has been rapidly gaining interest for veterinary medicine as a convenient and rapid diagnostic measure of disease status in pigs (5, 6). Oral fluid is composed of saliva and a transudate that originates from oral capillaries, particularly gingival crevicular fluid that leaks from the crevices between teeth and gum (7). This transudate is a product of the circulatory system and consequently contains many of the components found in serum, including antibodies (8–10).

Collecting OF samples from pigs using cotton ropes hanging in pens is an easy and welfare-friendly sampling method, relying on their natural chewing behavior and exploratory motivation (11, 12). The use of oral fluid is also attractive because sample collection does not require special training which makes samples easy to obtain. Moreover, the physical and biological risks associated with blood sampling are eliminated (13). Pigs chew the cotton ropes which absorb the OF. A rope thus contains a pooled sample, although the contribution of individual animals to the pool is unknown. Samples can then be assayed for the presence of specific antibodies indicating exposure to pathogens (14, 15). White et al. (15) showed that results obtained from a rope hung for 30–60 min in a pen 25/28 pigs were representative of 75% of the animals.

As there is a range of collection methods available, it is important to accurately describe the resulting samples using standardized terminology. Following the guidelines outlined by Atkinson et al. (16), *whole saliva* is defined as “the fluid obtained…by expectoration” and *oral fluid* as “the fluid obtained by insertion of absorptive collectors into the mouth.” Samples can be collected under stimulated and unstimulated conditions depending on the method of collection, or use of chemical stimulants to induce salivary flow (17). Samples collected with absorptive materials are often considered “stimulated” by masticatory action whereas samples obtained via expectoration or drooling are called “unstimulated” (16, 17).

The OF is collected under stimulated conditions, while the saliva is collected under unstimulated conditions.

Use of OF as an alternative to blood for the diagnosis and surveillance of important pathogens is of great interest in veterinary medicine due to the relative ease with which they can be obtained (11, 13). However, in order to be used as a routine surveillance tool, any developed or modified sample types need to be validated against current gold standard methods.

There are a range of commercially available ELISA kits for detection of exposure to bacterial pathogens, most of which are validated for use with serum, or meat juice (18). Such assays have the potential to be adapted to detect antibodies in oral fluid (19). When the test medium differs to that which the test kit was originally designed for, changes to the test protocol (for example, sample dilutions, incubation times and temperature) may be necessary to optimize the performance of the assay (20).

Several countries use serological surveillance to establish the prevalence of *Salmonella* pig herds as part of their national control programs (21, 22). ELISAs to detect anti-*Salmonell*a antibodies in serum and meat juice are used as an indicator for the degree of *Salmonella* burden in pig herds (23).

In this study, we adapted a commercial *Salmonella* ELISA kit (IDEXX Laboratories, Westbrook, ME USA) for use on pig saliva and OF samples. In order to evaluate the potential of oral fluids and saliva samples as alternative sample types, anti-*Salmonella* antibody responses in individual and pooled saliva and pen-based OF samples were compared with serum samples collected from the same animals. The results obtained from serum samples were used as a gold standard.

## Materials and Methods

### Sample Collection

This study was carried out in the United Kingdom in a farrow-to-finish farm consisting of approximately 500 sows and gilts, 2,000 weaners, 2,000 growers, and 2,000 finisher pigs. The farm involved in this study had experience of clinical disease in young animals associated with *Salmonella* serovar Typhimurium for many years. Individual paired blood and saliva samples (five samples from 20 pigs per pen, representing 25% of the pen population) were collected from four pens (A, B, C, and D; 10% of the total finisher boxes) of finisher pigs (Large White breed, approximately 17 weeks of age and 60–70 Kg). In addition, pooled OF samples were also collected from each pen by hanging a three-strand, twisted cotton rope following the method described by Prickett et al. (6). Cotton ropes were left in pens and collected after 30–40 min to allow approximately 75% of animals in the pen to chew the rope (15). No attractant was used.

Prior to sampling, pigs were marked in order to match the individual saliva and blood samples throughout the sampling process. Matched saliva and blood samples were taken from five pigs from each of the four pens. Blood samples were taken for veterinary diagnostic purposes, and any remaining serum was stored for use in this study. Individual saliva samples were collected from the buccal cavity using a cotton sponge (Salivette®, Sarstedt, Nümbrecht, Germany). Sponges were fixed to a sterile plastic rod and held in the mouth of the pigs until thoroughly moistened. After collection, the saliva sponges were placed in sterile tubes and chilled on ice for transport to the laboratory (<4 h). In order to gather a sufficient amount of saliva from each animal, two sponges were collected. The volume obtained from the two sponges was pooled and the saliva samples were first tested individually and then the remaining volume was used to create a pool from the five animals sampled in each pen.

To prevent cross-contamination, a new plastic rod and clean pair of gloves were used for each sample taken. At the laboratory, tubes containing saliva samples were centrifuged at 3,000 × g for 10 min and the supernatants stored at −80°C until testing (24, 25).

At the same time as the serum and saliva samples were collected, samples of pen-based (pooled) OF were collected from the same four pens. A three strand cotton rope of 12 mm of thickness and 50 cm long (RopeServices UK, Houghton Le Spring, UK) was suspended in each pen and left in place for 30–40 min. After being chewed by the pigs, each rope was manually squeezed and the OF placed in 50 mL sterile tubes and transported back to the laboratory in a cool box. All the OF samples were centrifuged (1,500 g for 10 min) and the supernatants stored in aliquots at −80°C until use (20).

For pen-based testing, pooled OF samples (cotton ropes samples) were collected with stimulation (by masticatory action) while individual saliva samples were collected without stimulation (no exogenous gustatory, or mechanical stimulation). Data from a previous bacteriological investigation of the farm's, reported 60% *Salmonella* prevalence in weaners pigs. Accordingly the pen-based sample size was calculated to detect *Salmonella* infection considering a minimum expected prevalence of 50% and 95% confidence level (26, 27). In addition to the farm samples collected, five individual saliva and three OF samples were collected from *Salmonella*-free sows housed in biosecure pens at the Animal and Plant Health Agency to serve as negative controls.

### Detection of *Salmonella*-Specific Antibodies by ELISA in Saliva, Serum, and of Samples

A commercial ELISA kit (IDEXX Swine *Salmonella* Ab Test, IDEXX Laboratories, Westbrook, ME, USA) validated for serum and meat juice samples was used to evaluate the presence of *Salmonella*-specific IgG antibodies in serum, saliva and OF samples.

Saliva and serum samples were tested individually and in pools. Saliva and serum pools were created using equal volumes of sample from each of the five animals sampled within a pen, resulting in four pools.

Individual/pooled serum samples were tested in duplicate, according to the manufacturer's protocol. Briefly, ELISA plates containing 100 μl samples diluted 1/20 were incubated for 30 min at 24°C, washed three times with wash buffer, then incubated for 30 min with 100 μl anti-porcine IgG conjugate. Plates were washed three times before incubation with 100 μl 3.3',5,5'-tetrametilbenzidine (TMB) substrate for 15 min. The reaction was then stopped by addition of 100 μl of stop solution. For each assay, positive and negative kit control samples were used. The absorbance values were read with a plate reader at 630 nm and the OD values converted into ELISA sample-to-positive (S/P) ratios to determine positive/negative result.

According to the manufacturer's instructions, samples with a S/P ratio above 1.00 were considered positive for *Salmonella*-specific IgG.

Individual and pooled saliva samples and pooled OF samples were also tested using the IDEXX ELISA kit. All samples were tested in duplicate using a modified protocol. Following a preliminary study using a range of dilutions (neat−1:8, results not shown), individual and pooled saliva samples and OF samples were diluted 1:1 in the dilution buffer. This dilution was the most effective in detecting differences between animals using minimum volumes of individual and pooled saliva samples and pooled OF samples.

Briefly, samples were diluted 1:1 and 50 μl added to wells which were incubated for an incubation time of 2 h at a temperature of 37°C. After this step, the protocol followed the one detailed for serum samples for completion of the assay. The five negative saliva samples and the three OF collected from *Salmonella*-free pigs were, respectively, pooled and included on each plate as a negative control. S/P ratio was calculated using the negative control serum of the kit.

### Statistical Analysis

Statistical analysis was performed using SPSS 25.0 (IBM SPSS Statistics, NY, US). Correlation analyses between ELISA S/P in saliva and serum (individual and pool) samples were performed using Spearman's *rho* ranked coefficient test. The positive or negative status of the individual saliva samples was compared to that of the matched serum samples. Cohen's *Kappa* coefficient was calculated to assess the agreement between saliva and serum samples. Values of *p* < 0.05 were considered statistically significant.

The receiver operating characteristic (ROC) curve was used to assess the optimal cut-off values for S/P) ratios interpretation of the saliva and OF results. Sensitivity (Se) and specificity (Sp) against the gold standard (ELISA examination of the sera) were calculated. The Kolmogorov–Smirnov test for goodness of fit was used to verify normality of the sample distribution, and, on the basis of the results of this test, the Mann–Whitney *U*-test and the Kruskal-Wallis H test were used to compare S/P values in sera and saliva samples at pen level and herd level, respectively.

## Results

Individual saliva samples were more difficult to obtain and needed to be collected in duplicate to obtain sufficient volume for testing. Sponges only yielded volumes of 467.2 ± 102 μl (mean ± SEM). One pig from pen A was omitted from testing as the saliva sponges yielded an insufficient sample. The volumes of two other saliva samples collected from pen A animals were only sufficient for testing individually and could not be used to contribute to a pool.

The volume of OF collected from hanging cotton ropes ranged from 3 to 8 ml per pen.

Significant differences were observed between S/P values in sera and saliva samples at herd level (all data together) and pen level.

**Figure 1:**
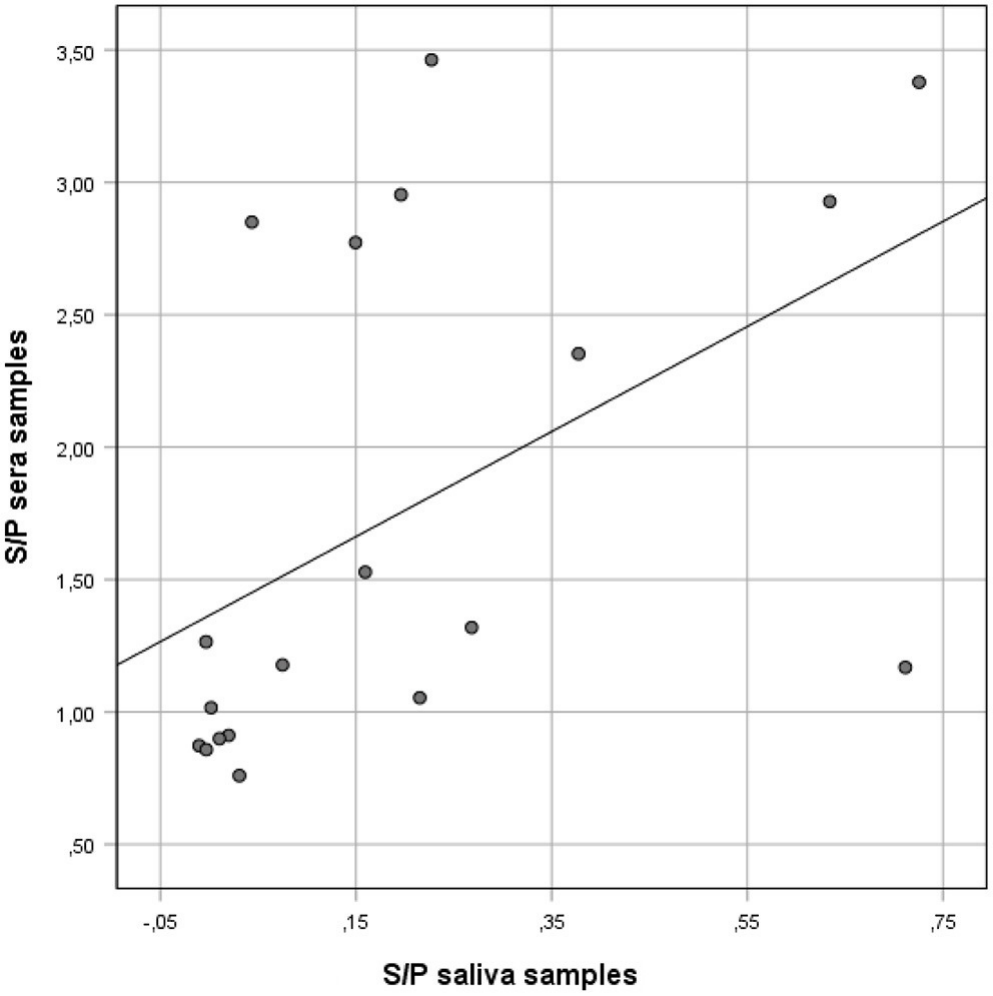
Correlation between anti-*Salmonella* ELISA IgG S/P ratio values of individual serum and matching S/P ratio saliva samples collected from finisher pigs. *Salmonella* IgG was detected on saliva and serum samples using a commercial ELISA kit validated for serum and meat juice.

At the herd-level the ELISA S/P ratio values for saliva samples were significantly lower than S/P values of the corresponding sera (*U* = 0.00; *p* < 0.001) (Figure 1 and Table 1). Similarly, significant differences were observed between S/P values of serum and saliva samples in each of the 4 boxes, with S/P values in sera always greater than the S/P values in the saliva samples (*U* = 0.00 *p* = 0.03; *U* = 0.00 *p* = 0.01; *U* = 0.00 *p* = 0.01; *U* = 0.00 *p* = 0.01 in pen A, B, C, D, respectively). No significant differences in S/P values for serum or S/P values saliva samples were observed between the four pens (*H* = 5.94; *p* = 0.12 and *H* = 2.87; *p* = 0.41, respectively).

**Table 1:** Anti-*Salmonella* ELISA IgG OD values of individual and pool samples of serum and saliva and pen-based OF.

**Pen**	**Animal**	**Individual serum**			**Individual saliva**			**Pool of serum**			**Pool of saliva**			**Pen based OF**		
		**OD**	**S/P ratio**	**Pos/Neg**	**OD**	**S/P ratio**	**Pos/Neg**	**OD**	**S/P ratio**	**Pos/Neg**	**OD**	**S/P ratio**	**Pos/Neg**	**OD**	**S/P ratio**	**Pos/Neg**
A	1	3.23	2.95	Pos	0.27	0.20	Pos									
A	2	1.25	1.17	Pos	0.76	0.71	Pos	0.79	0.67	Neg	0.09		Neg	0.35		Pos
A	3	1.15	1.05	Pos	0.29	0.22	Pos									
A	4	0.78	0.91	Neg	0.08	0.02	Neg									
B	6	2.82	2.85	Pos	0.10	0.04	Neg									
B	7	2.75	2.77	Pos	0.22	0.15	Pos									
B	8	3.39	3.46	Pos	0.30	0.23	Pos	2.63	2.45	Pos	0.32		Pos	0.54		Pos
B	9	1.26	1.18	Pos	0.15	0.07	Pos									
B	10	3.20	2.93	Pos	0.73	0.63	Pos									
C	11	0.98	0.87	Neg	0.07	0.00	Neg									
C	12	1.39	1.32	Pos	0.34	0.27	Pos									
C	13	3.32	3.38	Pos	0.83	0.73	Pos	2.19	2.03	Pos	0.33		Pos	0.35		Pos
C	14	1.59	1.53	Pos	0.23	0.16	Pos									
C	15	1.11	1.02	Pos	0.06	0.00	Neg									
D	16	0.87	0.76	Neg	0.09	0.03	Neg									
D	17	0.96	0.86	Neg	0.08	0.00	Neg									
D	18	1.00	0.90	Neg	0.09	0.01	Neg	1.37	1.23	Pos	0.28		Pos	0.38		Pos
D	19	1.25	1.26	Pos	0.05	0.00	Neg									
D	20	2.36	2.35	Pos	0.46	0.38	Pos									

*Positive samples are chosen in accordance with the S/P >1 for the serum and S/P>0.03 for saliva and OF samples. Serum and saliva pools of pen A were prepared by pooling only samples from animal 3 and animal 4*.

However, when the results of the two sample types were compared using Spearman's *rho* ranked coefficient, a positive correlation was observed (*rho* = 0.66; *p* = 0.002) (Figure 1).

The ROC curve analysis showed that the best correlation (Area under the curve, AUC: 90.0%) between saliva and serum ELISA results occurred when the saliva S/P ratio threshold was ≥0.03. Using the S/P ratio threshold ≥0.03 saliva samples had a Se and Sp of 86% (95%CL: 57–98) and 80% (95%CL: 28–99), respectively when compared with ELISA results obtained from individual serum samples (Table 2).

**Table 2:** Number of porcine serum and saliva samples positive and negative for anti-Salmonella IgG antibodies.

		**ELISA results in saliva (%)**		
		**Positive**	**Negative**	**Total**
ELISA results in serum	Positive	12 (85.7)	2 (14.3)	14
	Negative	1 (20.0)	4 (80.0)	5
	Total	11 (57.9)	8 (42.1)	19
		Se: 86% (95% CL: 57–98)	Sp: 80% (95% CL: 28–99)	*K*: 0.62

*Cohen's kappa coefficient showed a substantial agreement (K = 0.62)*.

Using Cohen's *Kappa* coefficient, a moderate agreement (*K* > 0.62 *p* = 0.002) was found between ELISA results for serum which represents the gold standard (positive if S/P ratio > 1.00) and saliva individual samples (positive if S/P ratio > 0.03). Only two seropositive pigs had saliva samples that yielded negative ELISA results.

In three of the four pens involved in this study, when individual samples were pooled the saliva and serum pools gave positive results even when positive samples were pooled with negative samples (pens C and D) (Table 1).

However, for pen A, only two individual samples, one positive and one negative (serum and paired saliva), were available to make the pool. In this case, saliva and serum pools were both negative.

Based on the sample size, a pen was defined as having a *Salmonella* seroprevalence ≥50% if at least one of the individual sera taken from that pen tested positive by ELISA.

Pen-based (pooled) OF data were analyzed and considered to be positive when the pen seroprevalence was ≥50%. Three of the four pens had a high proportion (>50%) of ELISA-positive sera and correspondingly OF collected from these pens tested positive for anti-*Salmonella* antibodies. In Pen D, despite the majority of the individual serum samples being negative, the OF sample collected from that pen was positive by ELISA.

## Discussion

In this study we modified the protocol of a commercial ELISA kit validated for serum and meat juice in order to test individual and pooled saliva samples (from oral sponges) and pen-based OF samples (from cotton rope chews) for the presence of anti-*Salmonella* antibodies in finisher pigs.

Although IgA is the predominant isotype present in OF (8, 9), several studies reported that IgG antibodies are a better target for determining exposure to specific pathogens (5, 28, 29). Compared with IgG, the IgA concentration seems to be more variably influenced by stress to the animals and by the rope material used for collection (5, 29). A previous study showed a lack of sensitivity for IgA detection in OF compared with the IgG isotypes (5). Therefore, only IgG levels where assessed in the current study.

Using a modified protocol (extended incubation time and increased temperature), we demonstrated that the IDEXX ELISA was able to detect anti-*Salmonella* antibodies in pig OF and saliva samples. Modifications to the sample dilution, incubation time and incubation temperature have significant effects on ELISAs to detect antibodies in OF (19, 30). Modifications of the original manufacturer's protocol were made to account for the lower concentration of antibody in OF and saliva samples. For this purpose, a decrease sample dilution was used and a longer sample incubation at high temperature was set up to allow potential antibody within the saliva and OF sample to bind to the antigen-coated on the ELISA plate. Modification of the ELISA was assessed, and Se and Sp were estimated at 86 and 80%, respectively, against the gold standard test (Table 2). Our study showed a moderate correlation between saliva and the corresponding serum results. This positive correlation indicates that the increase in S/P values of serum samples was correlated with an increase in S/P values saliva samples. These results suggest that individual saliva samples can represent a suitable alternative to blood samples for the detection of anti-*Salmonella* antibodies at an individual pig level.

Anti-*Salmonella* antibody levels in pig sera were always higher than in the matching saliva samples in all samples tested (*p* > 0.05). It has been reported that the IgG concentrations in OF are approximately 800 times lower than in serum (29). Therefore, pigs whose sera are only just above the ELISA cut-off could have saliva IgG levels below the limit of detection. Despite the substantial agreement found between individual serum and saliva samples, two seropositive pigs had saliva samples that yielded negative ELISA results in this study. These two negative results are not unexpected considering that the corresponding sera had S/P ratios only just above the ELISA kit cut-off, and similar variability has been found for meat juice when compared with serum (31).

By using pooled samples, a large number of animals may be analyzed for a reduced cost. However, it is important that the analytical performance of the assays remains high. Three pools were positive by ELISA, even when the pools consisted of positive and negative individual samples. However, for one pen (pen A) the dilution effect of pooling samples led to a loss of sensitivity, leading to a negative ELISA result. This could be due to the fact that for this pen only two of the five samples contributed to a pool. The risk of diluting positive samples with negative fluid to such an extent that the specific antibody concentration gives a negative ELISA result is a problem with pooling samples, but pooled samples are still suitable for herd screening unless the test sensitivity is very low (32–34). The effects of dilution depend on the relative concentrations of target antibodies in each sample.

Pen-based OF sampling using hung cotton ropes is another cost-saving strategy. The four OF samples collected by cotton ropes represented a pool of a higher number of animals compared with the five saliva samples collected individually.

Pen-based OF that originated from pens that had a high *Salmonella* seroprevalence (≥50) resulted to be ELISA-positive (Table 1) (26, 27). Even when the majority of the individual serum samples were negative (Pen D), the resulted OF sample tested positive for anti-*Salmonella* antibodies. This is presumably due to high levels of specific antibodies in the individual samples that were positive.

Despite the study was limited to one farm and a low number of samples were tested, to the best knowledge of the Authors, this is the first field study describing anti- *Salmonella* antibodies in pigs' OF. Although the results of this work should be evaluated with caution, we proved that the modification of the ELISA kit protocol allowed the detection of *Salmonella* IgG in saliva samples, emphasizing that this specimen has the potential to be used for the diagnosis of *Salmonella* infection in pig farms. Our work has demonstrated that individual saliva samples have the potential to be used for the diagnosis of *Salmonella* infection using the IDEXX ELISA with a modified protocol. Furthermore, pooled and oral fluid sampling using cotton ropes may have the potential for use in the detection of anti-*Salmonella* antibodies in field conditions.

Further studies are necessary to confirm and expand upon our findings. In particular, the effects of pooling, which is highly dependent on the dilution effect of mixing positive with negative samples, need to be fully understood. If there is great variability in antibody levels within the pen population, the strategy may lead to unreliable results. Furthermore, repeat sampling could lead to very different results.

The current study was carried out on a limited number of animals on a single farm. It is therefore recommended that further, larger scale studies are carried out in order to provide better evidence on the use of OF and saliva as a diagnostic samples for *Salmonella*.

## Data Availability Statement

All datasets generated for this study are included in the article.

## Ethics Statement

Ethical review and approval was not required for the animal study because the animals sampled in this study were undergoing veterinary investigation for a respiratory disease. The serum samples were collected by a veterinary surgeon on farm for diagnostic purposes, and therefore the collection was not a regulated procedure under the Animals (Scientific Procedures) Act (ASPA, 1986). The animals sampled were chosen for diagnostic purposes and the requirements of this study did not influence the selection of the animals or the volume of blood withdrawn. In this study excess serum was used if any was left after the sample had been used for diagnostic purposes. The collection of saliva samples or oral fluid samples does not constitute a regulated procedure under ASPA.

## Author Contributions

This study experiment was designed by AD, FO, and FM. AD: co-wrote paper and developed the study. SC: provided serological expertise at the laboratory level, co-wrote paper. RD and RS: provided critical review and revising of paper. CB: provided serological expertise and co-wrote paper. FO: performing statistical analysis and revising of paper. FM: developed study, and critically revised the manuscript. All authors read and approved the final manuscript.

### Conflict of Interest

The authors declare that the research was conducted in the absence of any commercial or financial relationships that could be construed as a potential conflict of interest.
